# Integrated multi-omics approach reveals novel associations in the rapeseed diet–microbiota–host axis in pigs

**DOI:** 10.1093/ismeco/ycae061

**Published:** 2024-04-23

**Authors:** Özgün C Onarman Umu, Liv Torunn Mydland, Chi Chen, Marta Pérez de Nanclares, Gerald C Shurson, Pedro E Urriola, Henning Sørum, Margareth Øverland

**Affiliations:** Department of Paraclinical Sciences, Faculty of Veterinary Medicine, Norwegian University of Life Sciences, Ås N-1432, Norway; Department of Animal and Aquacultural Sciences, Faculty of Biosciences, Norwegian University of Life Sciences, Ås N-1432, Norway; Department of Food Science and Nutrition, University of Minnesota, St Paul, MN 55108, United States; Department of Animal and Aquacultural Sciences, Faculty of Biosciences, Norwegian University of Life Sciences, Ås N-1432, Norway; Department of Animal Science, University of Minnesota, St Paul, MN 55108, United States; Department of Animal Science, University of Minnesota, St Paul, MN 55108, United States; Department of Paraclinical Sciences, Faculty of Veterinary Medicine, Norwegian University of Life Sciences, Ås N-1432, Norway; Department of Animal and Aquacultural Sciences, Faculty of Biosciences, Norwegian University of Life Sciences, Ås N-1432, Norway

**Keywords:** pigs, diet, microbiota, multi-omics, gut health

## Abstract

Diet-mediated host–microbiota interplay is a key factor in optimizing the gut function and overall health of the host. Gaining insight into the biological mechanisms behind this relationship is fundamental to finding sustainable, environment-friendly feed solutions in livestock production systems. Here, we apply a multi-omics integration approach to elucidate sustainable diet-associated host–gut microbiota interactions in pigs and we demonstrate novel and biologically relevant host–microbe associations in the gut, driven by a rapeseed meal-based feed (RSF). Interestingly, RSF-diet promoted the abundance of segmented filamentous bacteria *Candidatus Arthromitus* that was associated with the maintenance of mucosal immunity in the ileum of pigs. In the colon, RSF diet affected host mRNA splicing functions, which may result in different host gene products, through host–microbiota associations, particularly with the *Faecalibacterium* population, and through the interaction of dietary components such as sinapic acid with the host cells. Moreover, telomere maintenance and organization functions that may determine the overall health of the host were upregulated and notably associated with *Subdoligranulum* population in the colon of RSF diet-fed pigs*.* This integrative multi-omics approach provides more insight into the diet–microbiota–host axis, and a better understanding of mechanisms and opportunities to find new strategies for modulating host health and potentially improving caloric and nutritional efficiency in animal production.

## Graphical Abstract

**Graphical Abstract:**
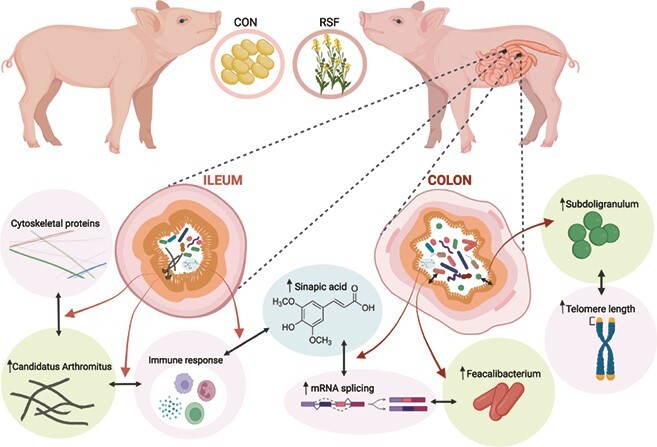

The most remarkable associations identified between bacterial groups or sinapic acid and host functional pathways in the ileum and colon of the rapeseed diet fed pigs, compared to control diet fed pigs. Bidirectional arrows indicate the associations between the variables (bacteria, sinapic acid, or functional pathways) identified by the multi-omics integration analysis. The upward arrows next to the variable names indicate the higher levels of these variables in ileum or colon of the RSF diet-fed pigs compared to the CON diet-fed pigs. Bacterial groups were shown in green circles, host functional pathways in purple circles, and sinapic acid in blue circle. Created by BioRender.com.

## Introduction

The future of food security and sustainability depends on integrating knowledge and implementing action plans from a macro-level (e.g. climate change mitigation) to a micro-level (e.g. systems biology to understand biological mechanisms). Systems biology is a rapidly emerging and essential omics platform for improving the efficiency of resource use to achieve long-term food security and sustainability. Multiple omics techniques (i.e. genomics, transcriptomics, proteomics, metabolomics) are being implemented in all areas of life sciences, including agriculture, to characterize the many complex interactions between genes, transcripts, protein, and metabolites [1]. In addition, multi-omics data can now be integrated to provide an even greater and more comprehensive understanding of the complex interactions and underlying mechanisms of biological responses to dietary interventions between the host and microbiota of food-producing animals [2]. Only a few research groups worldwide have effectively used this emerging “holo-omic” approach to determine host–microbiota interactions in basic and applied biological sciences [2].

Pork is one of the most widely consumed meat in the world [3], but the efficiency of utilizing dietary energy, protein (nitrogen), and phosphorus for producing carcass lean must be improved for sustainable pork production [4, 5]. Rapeseed (RS) is the second most abundant oilseed crop produced worldwide [6], and RS meal is often considered to be a more environmentally sustainable crop compared with soybeans, which is produced in much greater amounts. Improving the utilization of locally grown crops (i.e. RS) is one of many ways of reducing the environmental impacts of food production for greater long-term sustainability [7–9]. However, this can only be achieved if high nutritional efficiency of these renewable feed resources can be realized when they are added to diets of food-producing animals.

RS meal is an economical and abundant feed ingredient used primarily for its significant protein contribution in swine diets [10, 11]. However, it also contains significant amounts of dietary fiber and anti-nutritional factors that limit its nutritional value [10, 11]. We showed that feeding a RS co-product diet reduced the ileal and total tract digestibility of most nutrients and energy [12]. Using a metabolomics approach, we discovered that feeding RS meal to pigs caused a redox imbalance that was associated with reduced growth and nutritional efficiency [13]. We also discovered that feeding RS meal diets resulted in transcriptional responses in skeletal muscle and greater expression of genes responsive to oxidative stress [14]. Furthermore, feeding the RS meal diet to weaning and growing pigs resulted in more robust and beneficial gut microbiota populations and changes in the gut microbiota associated with enhanced potential for carbohydrate metabolism and reduced potential for bacterial pathogen pathways [15, 16]. However, we have not explored the host–microbiota interactions associated with feeding RS meal diets to young pigs using multi-omics data integration. In this study, we apply a multi-omics approach to explore the impact of feeding a RS meal-based diet on host–gut microbiota interactions in young pigs. For this purpose, we have analyzed datasets of microbiota from 16S rRNA sequencing, metabolomics, host gut transcriptomics, and host parameters using a multi-omics integrative approach that looks for a common information among these data types while discriminating between the dietary treatments. Hereby, we hypothesize that several novel diet-mediated associations between the gut microbiota and the host occur.

## Materials and methods

### Animals, dietary treatments, and sampling

The experimental design, procedures, diet formulation, methods, and sampling have been described in detail by Pérez de Nanclares *et al*. [12]. The research protocol was reviewed and approved by the Norwegian Food Safety Authority, and the experiment was conducted at the experimental farm of the Norwegian University of Life Sciences, Ås, Norway. Briefly, 40 Norwegian Landrace castrated male pigs (17.8 ± 2.7 kg) were fed either a control diet (CON) based on wheat, barley, and soybean meal (SBM), or an experimental diet (RSF) where wheat and SBM were partially replaced by high-fiber RS co-products (20 pigs/diet). The RS co-products consisted of 20% of a coarse fraction from air-classified RS meal and 4% of pure RS hulls [12]. The ingredient composition and nutritional value of the diets are presented elsewhere [12]. After 3 weeks of feeding, pigs were euthanized, digesta and tissue samples from the distal ileum and the apex of the spiral colon were collected, snap frozen, and kept at −80°C until DNA and RNA extractions. Ileum and colon samples from selected twelve pigs from each dietary treatment were used for the analyses. The randomized selection of the piglets was carried out based on their body weights, pens, and litters.

### 16S rRNA gene sequencing

The DNA extraction and 16S *rRNA* gene sequencing were performed as described in Umu *et al.* [15]. A subset of samples that were analyzed for microbiota profiles and published previously [15] were re-analyzed by using the amplicon sequence variant (ASV)-based approach. Basically, the paired-end demultiplexed raw reads were analyzed using DADA2 R package [17]. The fastq files were deposited in the Sequence Read Archive (SRA) (Bioproject ID: PRJNA427098 and Accession number: SRP127551). Filtering and trimming of the reads were performed using the parameters of truncLen 260 bp for forward reads and 250 bp for reverse reads, maxEE of 2 and truncQ of 2. The quality-filtered reads were dereplicated and denoised using the core DADA2 algorithm, and the paired reads were merged. The sequence table was constructed, and chimeras were removed. Taxonomy was assigned to the feature table using The RDP Classifier implemented in DADA2 R package with default settings using the Silva prokaryotic SSU taxonomic training data formatted for DADA2 with species (release-138.1, https://zenodo.org/record/4587955#.YIqLrLUzZPY). The feature table, taxonomy table, and metadata were imported as phyloseq object using phyloseq package [18] in RStudio. The feature table was filtered to keep the features that were present in at least 25% of the total samples and to remove the features that were assigned to Archaea, Chloroplast, and Mitochondria. The unclassified genera were agglomerated at and expressed with the lowest possible taxonomic group.

Supervised integration analysis of multi-omics data was conducted using the mixOmics R package [19]. Initially, pre-processing was performed based on the first step of the multivariate statistical framework mixMC [20]. An offset of one was applied to the whole dataset to deal with zeros, ASVs with low counts (cutoff = 0.01%) across all samples were filtered out and the Centered Log-Ratio (CLR) transformation was applied. The transformed data were processed for DIABLO N-integration analysis.

### Host parameters and metabolomics

Datasets of relevant host parameters were included in the integration analysis for ileum and colon separately (Table S1). Apparent ileal (AID) and total tract (ATTD) digestibility values were determined previously as described by Pérez de Nanclares *et al*. [12]. In addition, pH in ileum and colon, average daily feed intake, average daily gain, feed conversion ratio, and weight gain of pigs were measured, and blood serum parameters were analyzed at the Central Clinical Laboratory at the Norwegian University of Life Sciences (Ås, Norway). The morphometric analysis of Ki-67+ cells and crypt depth in colon was performed as described by Umu *et al*. [15].

The metabolomics analysis was previously performed and described by Chen *et al*. [13], using the liquid chromatography-mass spectrometry. The analyzed targeted metabolites included free AA, bile salts, volatile fatty acids, gluconapin, and sinapic acid from RS meal (Table S2).

### Transcriptomics

#### RNA extraction and quality check

Frozen samples of ileum and colon tissues (~0.5 × 0.5 cm) were homogenized, and the RNA in the samples was precipitated as described in the procedural guidelines of TRIzol reagent (Ambion, Carlsbad, CA) using 5 mm stainless steel beads (Qiagen, Hilden, Germany). Total RNA was purified using PureLink Pro 96 RNA Purification Kit (Invitrogen, Carlsbad, CA) according to the manufacturer’s instructions. The RNA was treated on-column with PureLink DNase (Invitrogen) to remove residual genomic DNA. Concentrations of RNA were measured by using a NanoDrop TM 8000 spectrophotometer (Thermo Fisher Scientific, Wilmington), and the RNA integrity value (RIN) was assessed by using a 2100 Bioanalyzer (Agilent Technologies, Waldbronn, Germany). All samples had a RIN above 8 and they were sent for sequencing at the Norwegian Sequencing Centre (Oslo, Norway).

#### Library construction, RNA sequencing, and data processing

A total of 48 RNA libraries (24 from ileum and 24 from colon, 12 per dietary treatment per tissue) were prepared for sequencing with TruSeq stranded mRNA prep (Illumina, San Diego) using unique barcodes, and 150-bp paired-end sequencing was performed across four lanes in a HiSeq 4000 System (Illumina, San Diego). Sequence data were processed with RTA v2.7.6 and it was demultiplexed and converted into fastq files using the unique bar codes with bcl2fastq v2.18.0.12. The fastq files were deposited in the SRA database (Accession number: PRJNA427098). The raw data from four lanes per sample were concatenated together before the analysis. Sequenced data were pre-processed by trimming reads containing adapter sequences, PhiX genome, and low-quality reads employing the BBDuK v34.56 set of the BBtools software package [21].

Trimmed sequences were aligned to the *Sus scrofa*11.1 genome assembly (Gene bank assembly accession GCA_000003025.6) with the tophat v2 [22] using the –library -type fr-firststrand—no-mixed—no-novel-junc options. The average insert fragment size for each sample was provided as parameter for the tophat alignment and it was calculated by aligning 1 million trimmed paired end reads against the *S. scrofa* cDNA using Bowtie v2.2.3 [23] and the Picard v1.112 CollectInsertSizeMetrics tool. The normalization of the data was performed by using DESeq2 (v1.26.0) [24].

### Multi-variate integration analysis

Data Integration Analysis for Biomarker discovery using a Latent component method for Omics studies (DIABLO) [25] was performed using the mixOmics R package v6.17.0 [19]. Due to the small sample size, the aim was to identify features across the datasets that were associated with the RSF diet and their correlations with each other, rather than to build a predictive model. Therefore, the *block.splsda()* function was used without specification of the *keepX* for this exploratory approach and five components were included in the model. The *perf()* function with leave one out (loo) cross-validation was used to assess the classification performance of the models for ileum and colon based on “weighted vote”, where the weight is defined according to the correlation between the latent component associated to a particular dataset and the outcome (www.mixomics.org) (Table S3). Receiver operating characteristic curve and area under curve (AUC) of the models were calculated for each block and each component (Figs S1 and S2) in addition to the combined AUC that was averaged across all blocks for each component (Table S4). Contribution of the variables from each data block to the dietary groups was identified by the model and visualized by loading plots based on the maximum level of expression. The contribution was interpreted as up- and downregulation of a variable in the categorical classes for a particular omics block, with the higher absolute values having more confidence.

The RSF diet-associated genes were detected (threshold of absolute importance value >0.03) based on the first component in the transcriptomics data block of the DIABLO model for both ileum and colon. Classification of the identified genes was performed using the g:GOSt tool in the g:Profiler web server [26]. Enrichment analysis was conducted for functional interpretation of the transcriptomics data by using the Gene Ontology (GO), Kyoto Encyclopedia of Genes and Genomes (KEGG) [27], and the Reactome [28] databases. Multiple testing correction was applied by using the tailor-made algorithm g:SCS of g:Profiler, which takes the hierarchical relation of the genes into account, and an adjusted *P*-value of ˂.05 was set as a cutoff. Some genes were associated with the bacterial populations and the primary components of RS, i.e. sinapic acid and gluconapin, according to the relevance networks. These genes were also subjected to the g:GOSt functional profiling to identify their interaction with the host cells. The gene identifiers (ID) were converted to gene names using g:Convert in g:Profiler, and the Ensembl gene ID was used where no gene name was available.

The correlation circle plot that highlights the contribution of each selected variable to each component, and the relevance networks that show the pairwise correlations were created based on a similarity matrix form DIABLO analysis to visualize the correlations between the different types of variables.

## Results

In our DIABLO model for integration of the datasets, a full-weighted design was used to optimize both discrimination of the dietary groups and correlation of the variables. The relative importance of the contribution of each variable to the dietary treatments was determined based on the first component of the model, which showed the highest discriminative power in both ileum (Fig. S3) and colon (Fig. S4). The correlation networks were created based on all five components involved in the model and uploaded to the Network Data Exchange (NDEx) Public Database (click for ileum network, click for colon network).

### Supervised integration analysis discriminated diet-associated bacteria, metabolites, and host parameters

#### Microbiota

**Figure 1:**
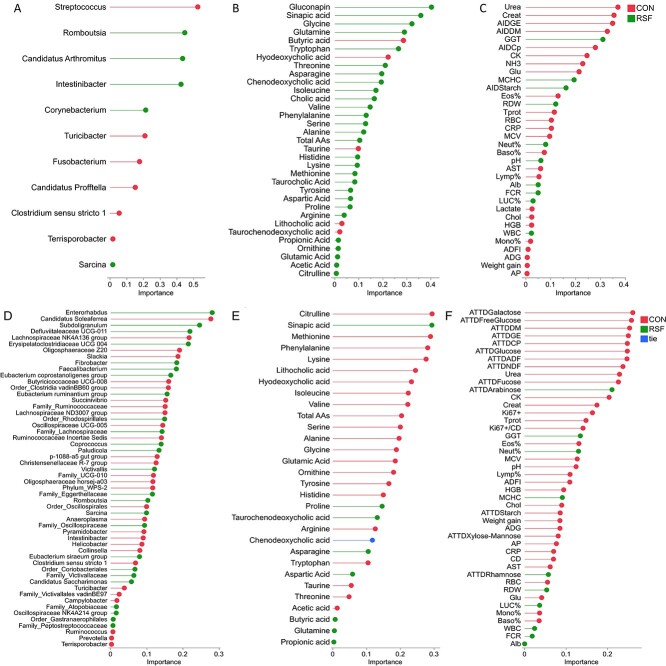
The loading plots of microbiome, metabolome, and host parameters in ileum content (A–C) and colon content (D–F) contributed to the first component of the DIABLO model. AID, apparent ileal digestibility; ATTD, apparent total tract digestibility, GE, gross energy; DM, dry matter; Cp, crude protein; ADF, acid detergent fiber; NDF, neutral detergent fiber; FCR, feed conversion ratio; ADG, average daily gain; CD, crypt depth; Ki67+, Ki67-positive cells; Creat, creatinine; CK, creatine kinase; Glu, glucose; MCHC, mean corposcular hemoglobin concentration; Eos %, eosinophils percentage; RBC, red blood cells; Tprot, total protein; CRP, C-reactive protein; MCV, mean corpuscular volume—erythrocytes; Baso%, basophils percentage; AST, aspartate aminotransferase; Lymp%, lymphocytes percentage; Alb, albumin; Chol, cholesterol; HGB, hemoglobin; mono%, monocytes percentage; AP, alkaline phosphatase.

After pre-filtering of low-count variables, 11 of 38 genera and 2118 of 31 907 genes remained in the ileum material, while 54 of 66 genera and 1990 of 31 907 genes remained in the colon material samples overall. These pre-filtered data with less sparsity were used for DIABLO N-integration analysis. The supervised integrative model identified weighted features from each dataset for each dietary treatment assigning an importance value (Fig. 1).

*Romboutsia*, *C. Arthromitus*, and *Intestinibacter* were identified to be more abundant in ileum in the RSF group with higher importance values, while *Streptococcus* was more abundant in the CON group with the highest importance (Fig. 1A, Table S5). In colon, *Enterorhabdus*, *Subdoligranulum*, *Defluviitaleaceae_UCG-011*, *Erysipelatoclostridiaceae_UCG-004*, *Fibrobacter*, and *Faecalibacterium* were among the taxa that were more abundant in the RSF group with higher importance values from the model (Fig. 1D, Table S5).

#### Metabolites and host parameters

Gluconapin in ileal digesta (not detected in colon) and sinapic acid in both ileum and colon were the metabolites with higher levels in the RSF group. Levels of the free AAs (except only taurine) were associated with the RSF diet in the ileum digesta, while levels of most of the AAs (except proline, asparagine, aspartic acid, and glutamine) in the colon were associated with the CON diet. Among the bile acids, levels of hyodeoxycholic acid, lithocholic acid, and taurochenodeoxycholic acid were higher in ileal digesta of the CON group, while the level of chenodeoxycholic acid was higher in the RSF group (Fig. 1B). In colon digesta, lithocholic acid and hyodeoxycholic acid were found in higher levels in the CON group and taurochenodeoxycholic acid in the RSF group, while chenodeoxycholic acid was not associated with any of the diet group particularly (assigned as tie in Fig. 1E). Among the volatile fatty acids in ileum digesta, the levels of acetic acid and propionic acid were higher in the RSF group with low importance values, and butyric acid was higher in the CON (Fig. 1B). On the other hand, acetic acid was higher in colon in the CON group, and butyric acid and propionic acid were higher in the RSF group with low importance values (Fig. 1E).

In concordance to our previously published results [12], both AID and ATTD of crude protein, dry matter, and gross energy were higher in the CON group, while AID of starch and ATTD of arabinose and rhamnose were higher in the RSF group. Levels of gamma-glutamyl transferase, mean corpuscular hemoglobin concentration, red blood cells distribution width (RDW), neutrophils percentage (Neut%), albumin, large unstained cells percentage (LUC%), and white blood cells (WBCs) in blood were associated with the RSF group (Fig. 1C and F).

#### Host transcriptome associated with RSF in ileum and colon of pigs

**Figure 2:**
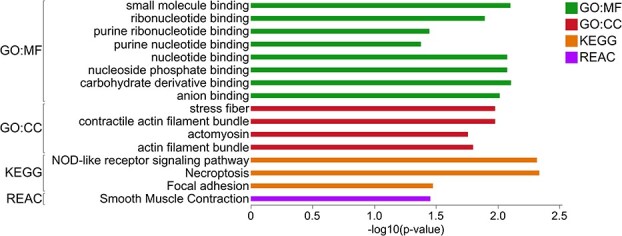
Functional profiling of enriched gene ontologies (GO), KEGG, and Reactome pathways in the ileum tissue of RSF-fed pigs; GO:CC, gene ontology cellular component; REAC, reactome pathways.

The genes discriminated for the RSF group (importance cutoff ≥0.03) (Tables S6 and S7) were subjected to enrichment analysis for functional profiling using the g:GOSt tool in the g:Profiler web server. In ileum tissue, 100 genes were upregulated in RSF group and the functional analysis of these genes resulted in “binding”-related enriched GO Molecular Functions (MFs) and “actin cytoskeleton”-related Cellular Components (CCs) (Fig. 2, Table S6). The enriched KEGG pathways in ileum of RSF-fed pigs included “NOD-like receptor signaling pathway”, “necroptosis”, and “focal adhesion”, and one Reactome pathway was enriched in ileum of the RSF group, which was “smooth muscle contraction”.

**Figure 3:**
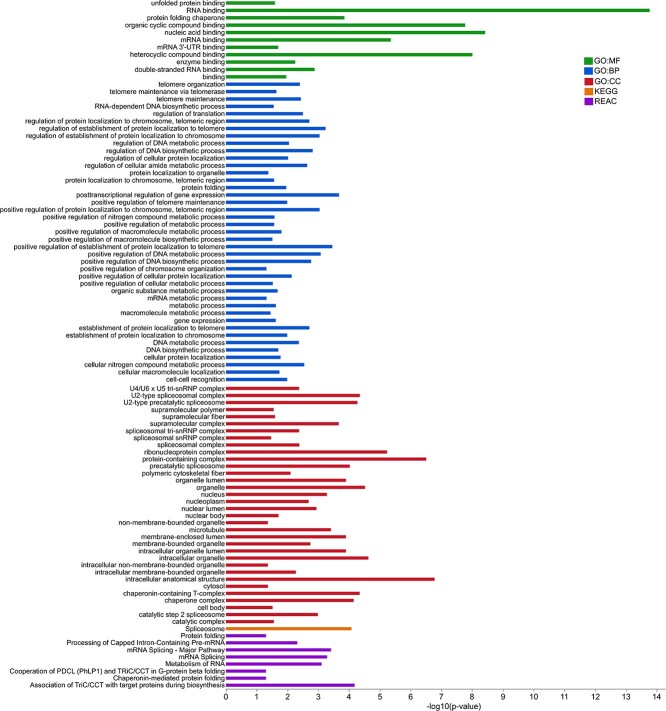
Functional profiling of enriched gene ontologies (GO), KEGG, and Reactome pathways in colon tissue of RSF-fed pigs; GO:CC, gene ontology cellular component; REAC, reactome pathways.

In colon tissue, 116 genes were upregulated in the RSF group (importance cutoff ≥0.03) (Table S7). The enriched GOs, KEGG, and Reactome pathways are shown in Fig. 3 and Table S7. Remarkably, GO biological processes (BPs) related to telomere organization and maintenance, DNA metabolic process, and cellular protein localization; MFs related to binding, supramolecular complex, and protein folding chaperone; CCs were related to the protein-containing complex and cellular anatomical entity; KEGG pathways of “Spliceosome”; and Reactome pathways of “Association of TriC/CCT with target proteins during biosynthesis”, “mRNA Splicing – Major Pathway”, “mRNA Splicing”, “Metabolism of RNA”, “Processing of Capped Intron-Containing Pre-mRNA”, “Chaperonin-mediated protein folding”, “Cooperation of PDCL (PhLP1) and TriC/CCT in G-protein beta folding”, and “Protein folding” were RSF-associated functions in colon of pigs.

### Data integration revealed diet-driven associations

**Figure 4:**
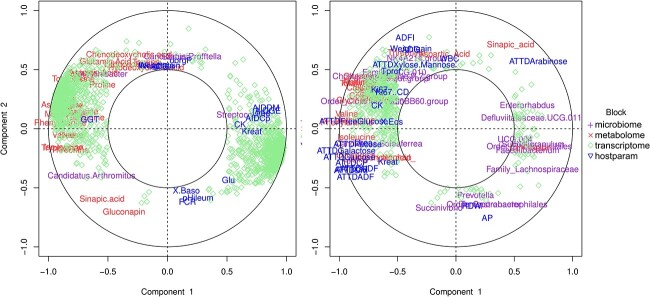
Correlation circle plots displaying contributions of the variables from all the blocks (datasets) on first and second components from the DIABLO models for ileum (A) and colon (B) with cutoff of 0.5 (inner circles); clusters of the variables indicate strong correlation between the variables.

We investigated the associations between features across the microbiota, metabolome, transcriptome, and phenotypic host response datasets according to the DIABLO models for ileum and colon separately. Diet-driven correlations were observed between the variables (Fig. 4). Most of the RSF-associated variables in ileum such as AAs, sinapic acid, gluconapin, and *Candidatus Arthromithus* clustered together showing strong positive correlations (on the left hand side of Fig. 4A), while CON-associated variables including *Streptococcus*, AID of dry matter, gross energy, and crude protein positively correlated with each other and negatively correlated with RSF-associated variables (projected at diametrically opposite places on the Correlation Circle [29]). This was also observed for colon, CON-associated variables mostly being situated on the left-hand side of the correlation circle plot (Fig. 4B) and RSF-associated variables on the right-hand side. The pairwise correlations between the different types of variables were further investigated by plotting relevance networks from the DIABLO models for ileum and colon (cutoff = 0.5) separately. The whole networks were uploaded on NDEx Public Database (click for ileum network, click for colon network).

### Diet-driven host–microbe association with biological relevance in ileum—*C. Arthromitus*, a distinctive bacterial group in ileum of the RSF group

**Figure 5:**
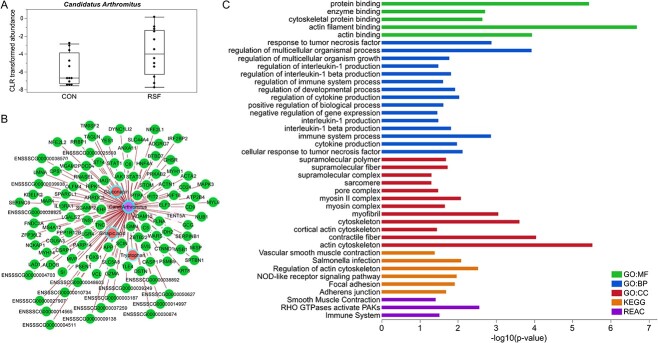
*C. Arthromitus* findings in ileum; (A) CLR transformed abundance of *C. Arthromitus* in CON and RSF samples; (B) relevance network of variables positively associated with *C. Arthromitus* in ileum; the color of node border indicates the importance of variable contribution, darker blue contributing in RSF group, darker pink in CON group; (C) enriched functional pathways of *C. Arthromitus*-associated genes, analyzed by using g:GOSt; GO:CC, gene ontology cellular component; REAC, reactome pathways.

The contributions of the microbiota populations to each diet group from the DIABLO model showed a higher abundance of *C. Arthromitus* in ileum of pigs fed RSF with high importance (Figs 1A and 5A). This bacterial population associated positively with the levels of sinapic acid, gluconapin, and tryptophan in ileum according to the relevance network (cutoff = 0.5), and negatively with the levels of creatine kinase, creatinine, urea, and the percentage of eosinophils in the blood, which were higher in the CON group (Fig. 1A). *C. Arthromitus* correlated positively with the host genes that are involved in cytoskeletal proteins and immune system-related functions (Fig. 5B and C), and most remarkably associated with IL-1beta production and cellular response to tumor necrosis factor among the functions related to immune responses (Fig. 5C).

### Microbiota-mediated associations of RSF diet with host genes in colon

**Figure 6:**
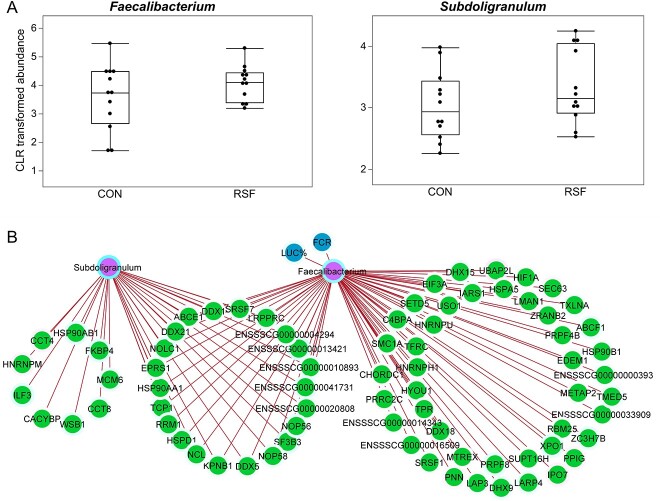
*Faecalibacterium* and *Subdoligranulum* findings in colon; (A) CLR transformed abundance of *Faecalibacterium* and *Subdoligranulum* in CON and RSF groups; (B) relevance network of variables positively associated with *Subdoligranulum* and *Faecalibacterium* in colon; the color of node border indicates the importance of variable contribution, darker blue contributing in RSF group, darker pink in CON group.

Among the bacteria with higher abundance in colon of the RSF group (Fig. 1D, importance >0.15 and Fig. 6A), *Subdoligranulum* and *Faecalibacterium*, both affiliated to the *Clostridia* class correlated positively with host genes (Fig. 6B), which were enriched most remarkably for GO BPs related to myeloid dendritic cell cytokine production and posttranscriptional regulation of gene expression. The whole list of functional pathways can be found in Table S8.

#### Telomere maintenance-related functions were enriched in colon tissue of the RSF group and highly associated with the *Subdoligranulum* genus

**Figure 7:**
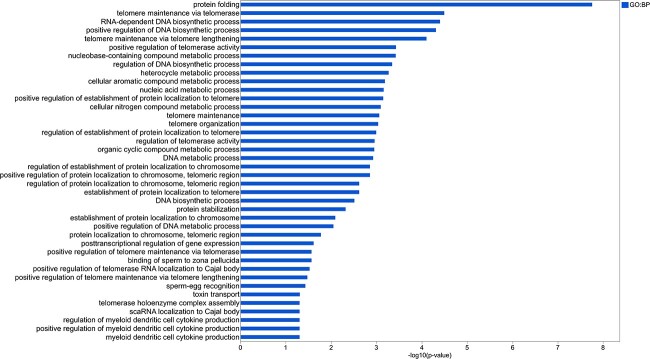
Enriched GO BPs in *Subdoligranulum*-associated genes.

The host genes in colon that correlated with *Subdoligranulum* (Fig. 6B) were submitted to g:Profiler for functional profiling using g:GOSt tool. Many GO BPs related to telomere organization and maintenance, including those found to be upregulated in colon of RSF group (Fig. 3), were associated with the *Subdoligranulum* genus (Fig. 7). The other GO BPs enriched in genes positively correlated with *Subdoligranulum* were related to myeloid dendritic cell cytokine production and metabolic processes including nucleic acid metabolic processes (Fig. 7). All the pathways enriched for *Subdoligranulum*-associated host genes are provided in Table S8.

#### *Faecalibacterium* associated with alternative mRNA splicing, gene expression, and regulation of translation functions in colon

Functional profiling of the host genes positively correlated with *Faecalibacterium* in colon resulted in, most remarkably, enrichment of GO BPs and Reactome pathways related to RNA processing, mRNA splicing, gene expression, and regulation of translation. All the pathways enriched for *Faecalibacterium*-associated host genes are provided in Table S8. These GO BPs did not enrich in the discriminated genes for RSF diet; however, Reactome pathways “mRNA Splicing - Major Pathway” and “mRNA Splicing”, and GO MFs, “RNA binding” and “mRNA binding” were also discriminated by the diet, associated with RSF group (Fig. 3).

### Sinapic acid associated with host functional pathways related to the immune system process in ileum and mRNA splicing in colon

**Figure 8:**
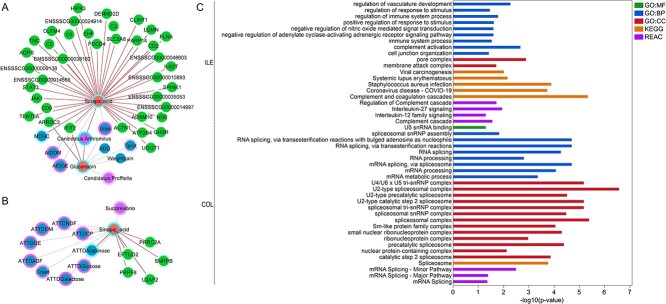
Sinapic acid and Gluconapin findings in ileum and colon; (A) relevance network of bacteria, host parameters, and positively associated genes with sinapic acid and gluconapin in ileum; (B) relevance network of bacteria, host parameters, and genes positively associated with sinapic acid in colon; the color of node border in the networks indicates the importance of variable contribution, darker blue contributing in RSF group, darker pink in CON group; (C) enriched functional pathways in genes positively associated with sinapic acid in ileum and colon (from A and B); ILE, ileum; COL, colon; GO:CC, gene ontology cellular component; REAC, Reactome pathways; the whole networks for ileum and colon were uploaded on NDEx Public Database (click for ileum network, click for colon network).

According to the relevance networks from DIABLO analysis, sinapic acid associated with host genes in ileum that were enriched for GO:CC of “membrane attack complex”; GO:BPs related to the immune system, namely “immune system process”, “regulation of immune system process”, “complement activation”; and GO:BPs of “regulation of vasculature development”, “positive regulation of response to stimulus”, “negative regulation of nitric oxide mediated signal transduction”, “negative regulation of adenylate cyclase-activating adrenergic receptor signaling pathway”, and “cell junction organization” (Fig. 8A and C). The KEGG pathway of “Complement and coagulation cascades” and the Reactome pathways of “Interleukine-27 signaling”, “Interleukin-12 family signaling”, and “regulation of complement cascade” were among the other functional profiles of the genes associated with sinapic acid in ileum. On the other hand, mRNA and RNA splicing-related GO:BPs and Reactome pathways, and spliceosome-related GO:CCs and KEGG pathways were enriched in genes that were associated with sinapic acid in colon of pigs (Fig. 8B and C).

## Discussion

Understanding biological mechanisms behind the diet-mediated host–microbiota crosstalk is an overarching goal to modulate and improve the gastrointestinal functions and overall well-being of the host. It is also important to promote the use of locally produced, sustainable feed ingredients in the diet of food-producing animals, and ultimately reduce the environmental impact of food production for long-term sustainability. We applied a multi-omics approach to elucidate the host–microbiota interactions associated with diet containing RS co-products in young pigs and found RSF-diet-driven novel and biologically relevant host–microbe associations.

We used an exploratory approach rather than a predictive one in our DIABLO integrative classification model [25] as a precaution due to the relatively low number of samples (*n* = 12 per dietary treatment). Nevertheless, biological interpretation of the discriminated features for diets showed consistency with our previous findings using different statistical methods for the whole sample set, such as lower digestibility values in RSF pigs [12], and association of amino acids with RSF diet in ileum and with CON diet in colon [15]. We used the ASV method and the Silva taxonomy database for microbiota analysis prior to integration. Similar bacterial groups were discriminated by the DIABLO model, compared with our previous operational taxonomic unit (OTU)-based analysis using the Greengenes database [15]. However, the resolution of taxonomic classification by the ASV method in this analysis was higher. On the other hand, we also detected new bacterial taxa discriminated for the RSF group. Interestingly, the *C. Arthromitus* group that has not been identified or correctly classified based on the OTU approach previously [15] was detected in ileum of piglets, while was found to be absent in colon. *C. Arthromitus* affiliated to the *Clostridiaceae* family, is a proposed candidate of segmented filamentous bacteria and was found to be predominant in ileum of RSF-fed piglets. Segmented filamentous bacteria are commensal organisms that attach to the ileal epithelium of the host without any inflammatory response, they rather play a key role in intestinal immunity and homeostasis to establish normal gastrointestinal functions [30, 31]. Among a large number of animal species as hosts, segmented filamentous bacteria are present in pigs, especially abundant in ileum and cecum of post-weaning piglets [32, 33]. As a key player in host–microbial interactions of the gastrointestinal tract, *C. Arthromitus* associated with genes in the ileal epithelium of piglets that are involved in cytoskeletal proteins and immune system-related functions. Segmented filamentous bacteria are known to induce focal displacement of the microvillar brush border, and actin polymerization occurs similar to the active host cellular response to *Salmonella typhimurium* infection as they both attach to the same site in the host [31]. This explains the association of *C. Arthromitus* with “Salmonella infection” and regulation of actin cytoskeleton KEGG pathways as well as GO:CCs of cytoskeleton protein in our study. Furthermore, *C. Arthromitus* correlated positively with the host genes enriched for cytokine production GO BP in ileum, particularly interleukin 1 (IL-1) and interleukin 1 beta (IL-1β) production, as well as cellular response to tumor necrosis factor. These cytokines play role in differentiation of Th17 cells, such that coordinated activity of IL-1 and TNF can accelerate the process [34] and induction of IL-1β by commensal microbiota is essential for the development of Th17 cells [35]. Our findings are in concordance with studies showing that segmented filamentous bacteria and their flagellins induce intestinal Th17 cells, which are critical for maintenance of mucosal immunity [36, 37]. Therefore, we speculate that microbiota-mediated regulation of immunity in ileum of pigs may occur when they are fed high-fiber RSF diet. However, production of these cytokines and effect on Th17 cell differentiation in ileum of pigs fed RSF diet needs validation with further studies. Moreover, in the ileum, sinapic acid associated with immune-related functions, including the GO:CC of membrane attack complex (MAC), which is a pore complex and an important innate immune effector of the complement terminal pathway [38]; GO:BPs related to immune system, namely “immune system process”, “regulation of immune system process”, and “complement activation”; and Reactome pathways of “Interleukine-27 signaling”, “Interleukin-12 family signaling”, and “regulation of complement cascade”. Nevertheless, sinapic acid is known to confer an anti-inflammatory effect, and one of the mechanisms is known to be suppression of lipopolysaccharide-induced nitric oxide [39]. Concordantly, we found association between sinapic acid and “negative regulation of nitric oxide mediated signal transduction” pathway in ileum.

Host genes in the colon of the RSF group were enriched for the telomere length and maintenance functions, which were highly associated with the *Subdoligranulum* population. Telomere length is highly correlated with aging, and shorter telomeres are associated with higher risk of disease and mortality. Therefore, preserving telomere length plays an important role in ensuring health [40]. Dietary fibers may play a role in maintenance of telomere length in colon, a study reported that inclusion of resistant starch in human diet attenuated the colonocyte telomere shortening that increased by meat consumption and showed protective effect against colorectal cancer risk [41]. Microbiota predominated by commensal bacteria has recently been reported to result in longer telomeres in bird hatchlings, highlighting the microbiota effect on telomere maintenance and the importance of early establishment of microbiota for health and survival [42]. The extensive analysis of metabolic response of the piglets to the RSF diet performed by Chen *et al*. for the same pigs in our study [13] has identified a redox imbalance in metabolome of the RSF piglets compared to the CON. Numerical studies have shown that oxidative stress is a major factor contributing significantly to telomere shortening [43]. Our integration analysis findings were contradictory since the RSF diet upregulated the genes enriched for telomere maintenance and organization pathways in colon of pigs; moreover, these functions were highly associated with the RSF diet-enriched commensal bacterial group *Subdoligranulum* belonging to the *Clostridium* cluster IV according to the integration analysis. Therefore, we hypothesize that there could be a dynamic regulation, elucidated by our multivariate integration analysis and probably occur constantly, and that the oxidative stress caused by the RSF diet is counteracted by the microbiota supported by the same diet. The length of telomeres in colon was not measured in our study as this association is a novel finding and was not expected. However, these specific, interesting correlations indicating the diet-mediated host-microbiota interaction merits further investigation.

There is a close interaction between microbiota, gene regulation, and splicing architecture of the host mucosal transcriptome, and precise nature of this interaction is needed for the intestinal homeostasis [44]. Microbiota–host gene expression cross-talk can occur via different mechanisms, such that the gut microbiota remodels host chromatin, causes differential splicing, alters the epigenetic landscape, and directly interrupts host signaling cascades [45]. Our study illustrated the possible role of commensal bacteria *Faecalibacterium*, which was predominant in the RSF group, in host gene regulation in colon via alternative splicing of the transcriptome. These functions were also found to be enriched for discriminated genes in the RSF group, suggesting diet-associated microbe–host gene interaction. In addition, sinapic acid levels in colon positively correlated with five genes U2AF2, EFTUD2, PRRC2A, SNRPB, and PRPF8 that were enriched for mRNA splicing and spliceosomal complex-related functional pathways, indicating association of dietary components with the host gene regulation. Notably, Eftud2 is an mRNA splicing regulator and it has been reported to modulate the innate immune response through controlling the alternate splicing of the MyD88 innate immunity signaling adaptor [46]. This may suggest a role of sinapic acid as an additional layer of immunity modulation mediated by mRNA splicing.

Our integration analysis identified a positive association of feeding the RSF diet with some host blood parameters, including RDW, Neut%, albumin, LUC%, WBC, GGT, and MCHC. Association of RSF with RDW, Neut%, LUC%, and WBC in blood may indicate that the immune system is activated to a larger extend in the RSF group than in the CON group. And the higher level of GGT in RSF group may point out that the compounds in RS may cause minor toxic damages in liver cells causing leakage of GGT. On the other hand, the levels of all these metabolites were within the normal range for this age of piglets, and there was no difference in the hematology between the dietary groups [12]. Therefore, the slightly activated immune activity, in concordance with the host gene expression findings, indicates the enhancement of immune function of piglets in RSF group.

In conclusion, our study revealed novel positive microbiota–host associations that were mostly mediated by the RSF diet. Higher concentrations of dietary fibers enriching specific bacterial groups and sinapic acid in the RSF diet were found to affect host gene expression. Functional pathways in the gut related to the immune system, telomere maintenance, and mRNA splicing were highlighted to be linked to the microbiota-mediated effect of the RSF diet. *C. Arthromitus* in ileum and *Faecalibacterium* and *Subdoligranulum* in colon were the most notable bacterial populations enhanced by feeding the RSF diet and associated positively with these host functional pathways. The metataxonomics approach followed for the microbiota analysis in this study has revealed novel findings; on the other hand, a metagenomics approach or a combination of these amplicon and genome sequencing approaches would increase the resolution at lower taxonomics ranks and provide new insights on the host–microbiome associations. It is also noteworthy that the direction of causality with these associations is unknown and assessment of the direction of these two-way conversations with host gene regulation and validation of specific correlations as well as host gene expressions remain to be further investigated. Using multi-omics approach, we found novel associations in the diet–microbiota–host axis in pigs when fed a sustainable RS-based diet. Deciphering the biological mechanisms using such an integration approach will increase the knowledge in host–microbiota interactions and awareness in livestock production systems and contribute to animal health and welfare and ultimately the future of sustainable food security.

## Supplementary Material

Supplementary_Information_revised_ycae061

Table_S1_ycae061

Table_S2_ycae061

Table_S3_ycae061

Table_S4_ycae061

Table_S5_ycae061

Table_S6_ycae061

Table_S7_revised_ycae061

Table_S8_ycae061

## Data Availability

The datasets analysed during the current study are available in the Sequence Read Archive (SRA) (Bioproject ID: PRJNA427098 and Accession number: SRP127551), https://www.ncbi.nlm.nih.gov/sra/?term=SRP127551.
