# Supplementary material for: Integrated multi-omics approach reveals novel associations in the rapeseed diet–microbiota–host axis in pigs
Source: ISME Commun. 2024 Apr 23;4(1):ycae061. doi: 10.1093/ismeco/ycae061 (PMC11128262; doi:10.1093/ismeco/ycae061)
Supplement: Supplementary_Information_revised_ycae061 [file supplementary_information_revised_ycae061.docx]

**DIABLO Model and Plot Interpretations**

In DIABLO, latent components (linear combinations of variables) are built with the goal of maximizing the sum of covariances between all pairs of datasets. These latent components are defined by their corresponding loading vectors, which are vectors with the weight of each original variable's contribution to the corresponding latent component. Greater absolute values in this loading vector means that a given variable has a greater “importance”. The importance values in **Figure 1** have been calculated as described above.

The “-log10(p-value)” in **Figure 2, 3, 5, 7 and 8** were calculated by the g:GOSt tool in the g:Profiler web server for the functional enrichment analyses. The detailed output table and g:Profiler link for these analyses and figures can be found in supplementary tables as indicated in the manuscript.

The correlation circle plot in **Figure 4** shows the correlation between each of the variables in the model. Close proximity of the variable vectors to one another indicates a positive correlation. A positive correlation is shown by an acure angle (smaller than 90°) between the two vectorsand a negative correlation is shown by an obtuse angle (more than 90°). Their correlation is zero if they are at a straight angle. So, the cosine of the angle formed by the vectors of two features (which start at the origin) determines their correlation.

The relevance networks in **Figure 5, 6 and 8** visualize the correlation between the different types of variables pairwise and also built on the similarity matrix as the correlation circle plots does.

The interpretation of all the graphics from the mixOmics package is described detailly on its website <http://mixomics.org/graphics/>.

**A**

| **Block: Microbiome** | | |
| --- | --- | --- |
| **CON vs RSF** | **AUC** | **p-value** |
| Comp1 | 0.7708 | 0.02434 |
| Comp2 | 0.8681 | 0.00221 |
| Comp3 | 0.8819 | 0.0015 |
| Comp4 | 0.8889 | 0.00122 |
| Comp5 | 0.9028 | 0.00081 |
|  |  |  |
| **Block: Metabolome** | | |
| **CON vs RSF** | **AUC** | **p-value** |
| Comp1 | 0.8819 | 0.0015 |
| Comp2 | 0.7431 | 0.04331 |
| Comp3 | 0.7569 | 0.03266 |
| Comp4 | 0.7569 | 0.03266 |
| Comp5 | 0.7639 | 0.02824 |
|  |  |  |
| **Block: Transcriptome** | | |
| **CON vs RSF** | **AUC** | **p-value** |
| Comp1 | 0.7083 | 0.08326 |
| Comp2 | 0.9236 | 0.00043 |
| Comp3 | 0.9444 | 0.00022 |
| Comp4 | 0.9931 | 4.15E-05 |
| Comp5 | 1 | 3.23E-05 |
|  |  |  |
| **Block: Host parameters** | | |
| **CON vs RSF** | **AUC** | **p-value** |
| Comp1 | 0.8958 | 0.001 |
| Comp2 | 0.7986 | 0.01304 |
| Comp3 | 0.7986 | 0.01304 |
| Comp4 | 0.7986 | 0.01304 |
| Comp5 | 0.7986 | 0.01304 |


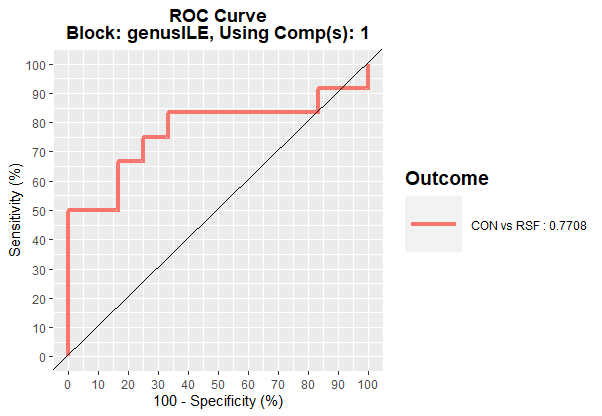


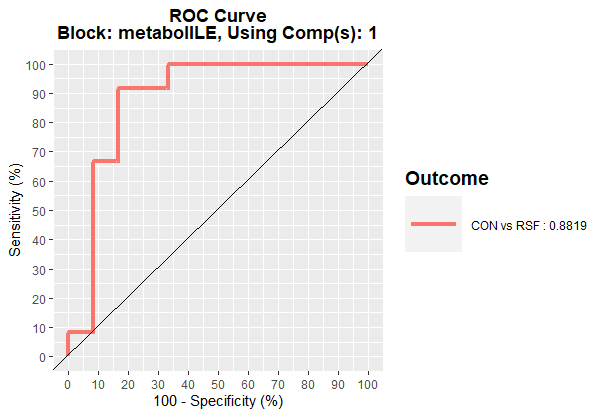


**B**

**C**


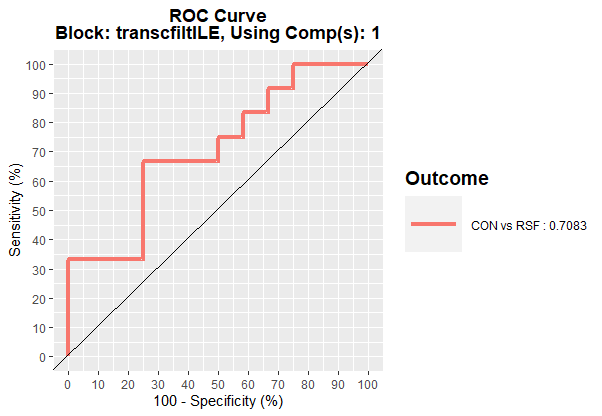


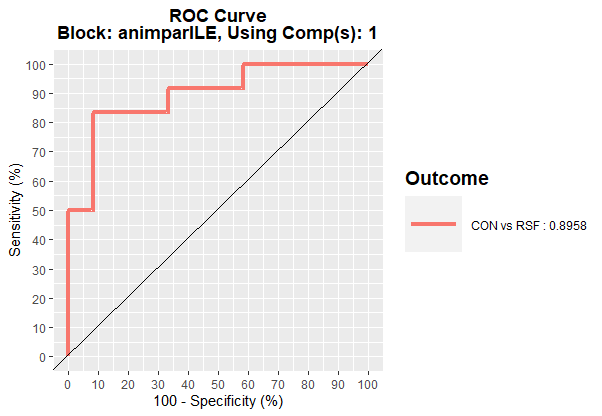


**D**

**Figure S1**. The ROC curves for the first component of the ileum DIABLO model and the area under curve (AUC) values calculated for each block and each component. A: Microbiome, B: Metabolome, C: Host transcriptome, D: Host parameters. P-values were calculated from a Wilcoxon test between the predicted scores between one class vs the other (CON vs RSF)

| **Block: Microbiome** | | |
| --- | --- | --- |
| **CON vs RSF** | **AUC** | **p-value** |
| Comp1 | 0.8264 | 0.006657 |
| Comp2 | 0.9861 | 5.31E-05 |
| Comp3 | 0.9931 | 4.15E-05 |
| Comp4 | 0.9931 | 4.15E-05 |
| Comp5 | 0.9931 | 4.15E-05 |
|  |  |  |
| **Block: Metabolome** | | |
| **CON vs RSF** | **AUC** | **p-value** |
| Comp1 | 0.7569 | 0.03266 |
| Comp2 | 0.7708 | 0.02434 |
| Comp3 | 0.7639 | 0.02824 |
| Comp4 | 0.7986 | 0.01304 |
| Comp5 | 0.8333 | 0.005584 |
|  |  |  |
| **Block: Transcriptome** | | |
| **CON vs RSF** | **AUC** | **p-value** |
| Comp1 | 0.8472 | 0.003892 |
| Comp2 | 1 | 3.23E-05 |
| Comp3 | 0.9931 | 4.15E-05 |
| Comp4 | 1 | 3.23E-05 |
| Comp5 | 1 | 3.23E-05 |
|  |  |  |
| **Block: Host parameters** | | |
| **CON vs RSF** | **AUC** | **p-value** |
| Comp1 | 0.8681 | 0.002214 |
| Comp2 | 0.9653 | 0.00011 |
| Comp3 | 0.9514 | 0.000175 |
| Comp4 | 0.8958 | 0.000999 |
| Comp5 | 0.8889 | 0.001224 |


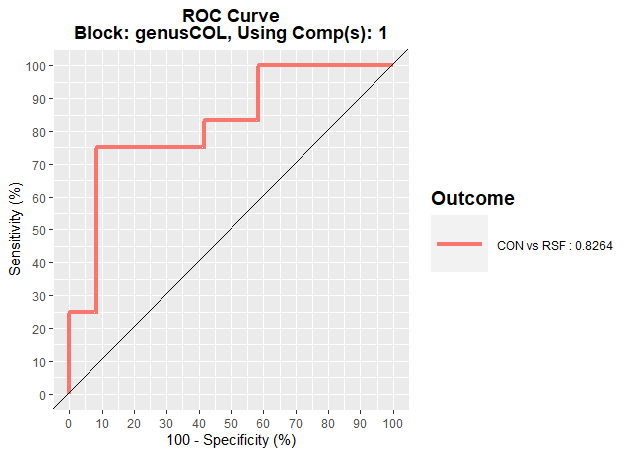


**A**

**C**

**B**


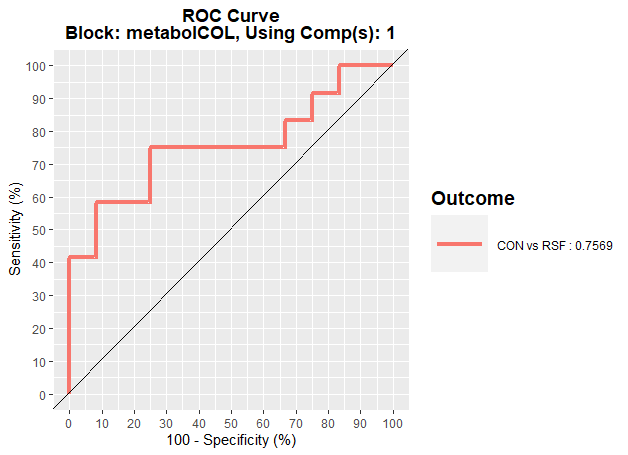


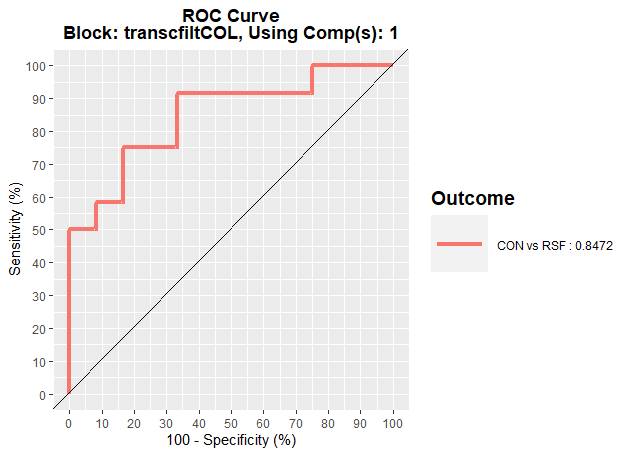


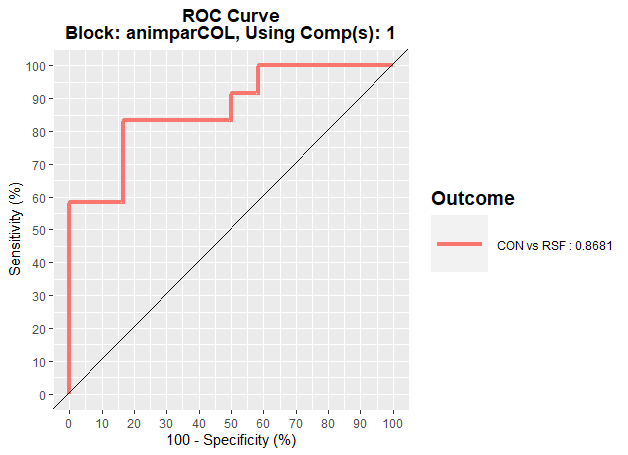


**D**

**Figure S2**. The ROC curves for the first component of the colon DIABLO model and the area under curve (AUC) values calculated for each block and each component. A: Microbiome, B: Metabolome, C: Host transcriptome, D: Host parameters. P-values were calculated from a Wilcoxon test between the predicted scores between one class vs the other (CON vs RSF)


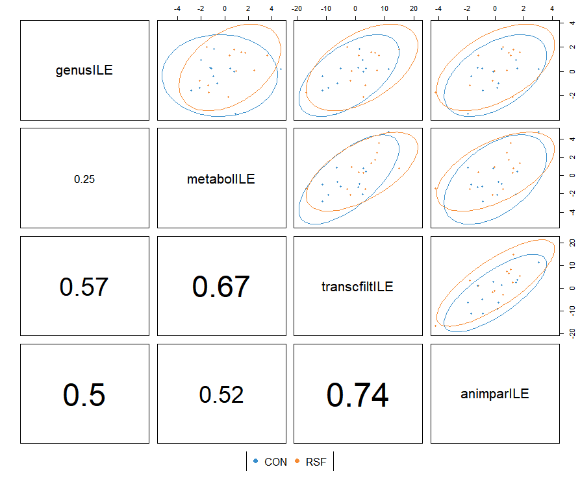

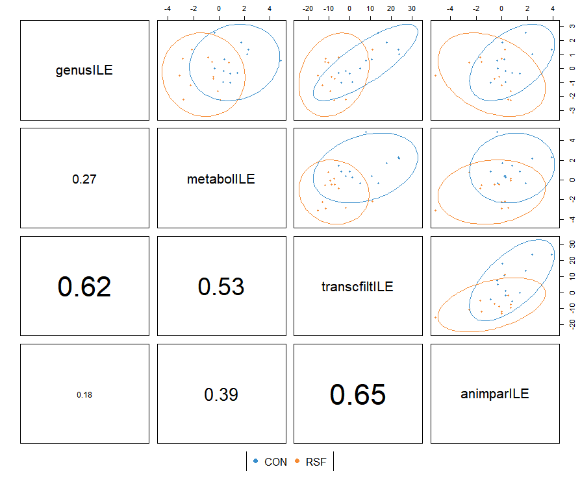

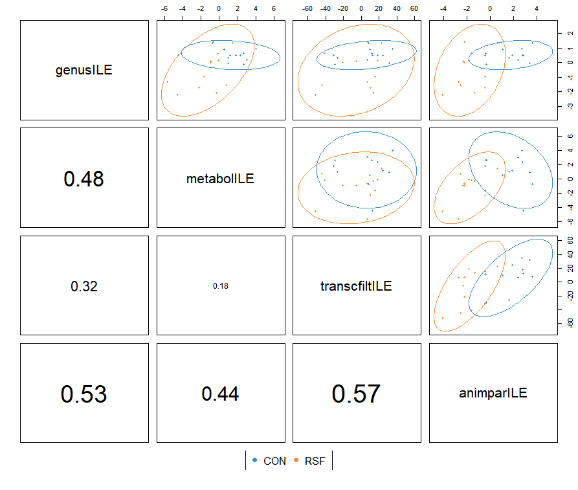


C

B

A


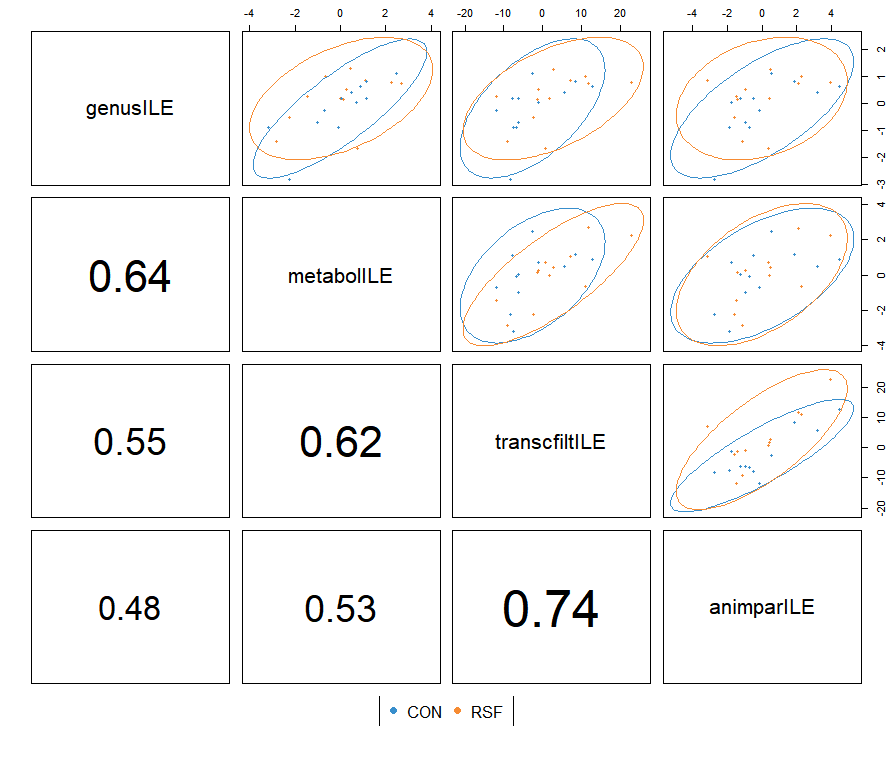


D


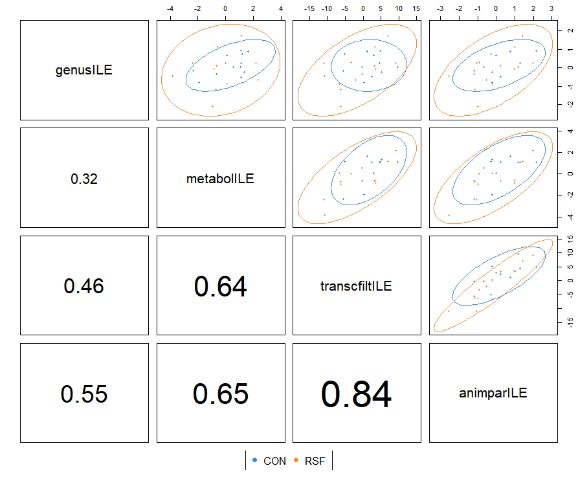


E

**Figure S3.** Diagnostic plot from the Diablo model applied on the ileum samples. The samples are represented based on the 1^st^ (A), 2^nd^ (B), 3^rd^ (C), 4^th^ (D) and 5^th^ (E) component of the model. The samples are colored by the diet type and 95% confidence ellipse plots were shown. The numbers indicate the correlation between components from each data set, and the colors and ellipses indicate the discriminative power of each component to separate CON and RSF groups. genusILE: microbiome data; metabolILE: metabolome data; transcfiltILE: transcriptome data; animparILE: animal parameters data from the ileum samples.


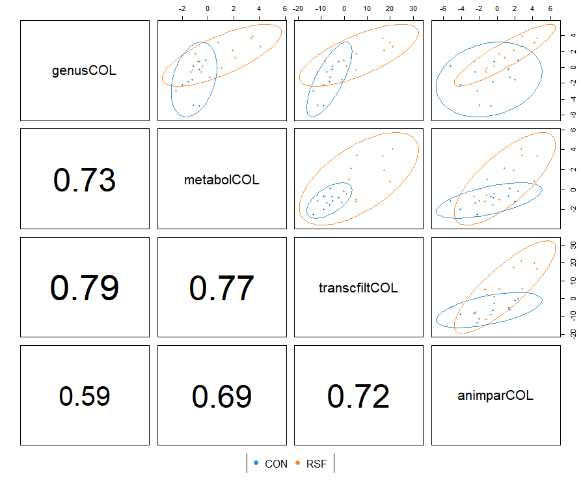

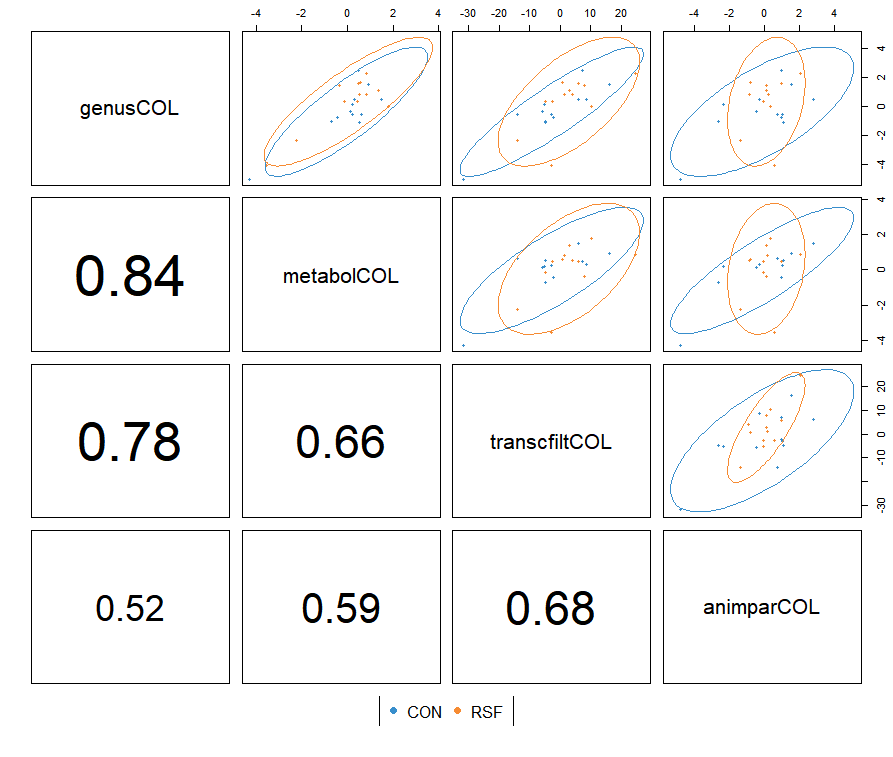

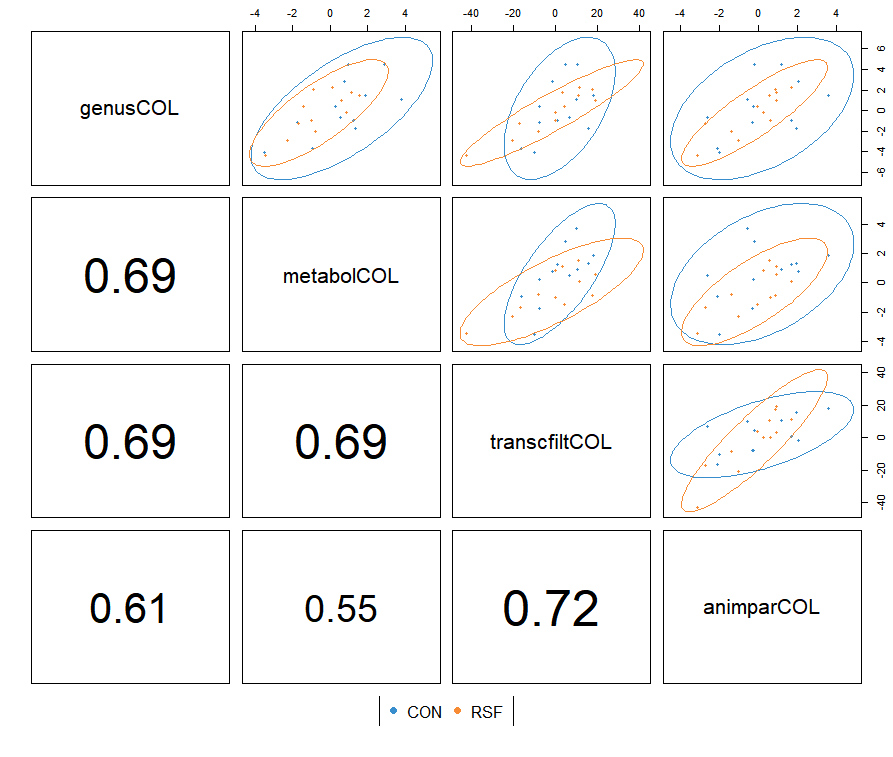

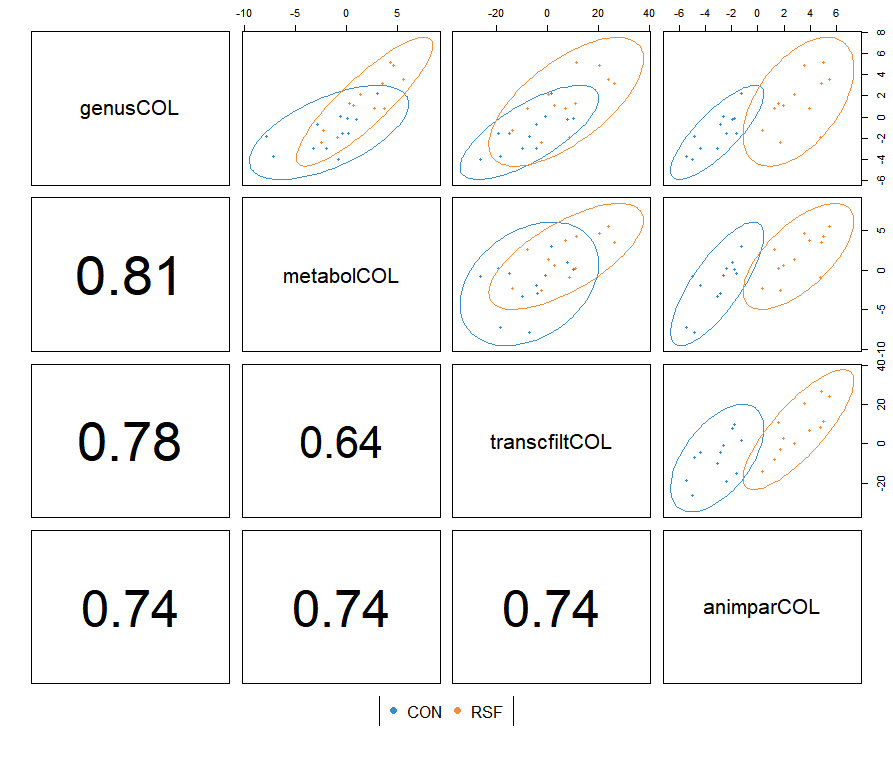


D

C

B

A


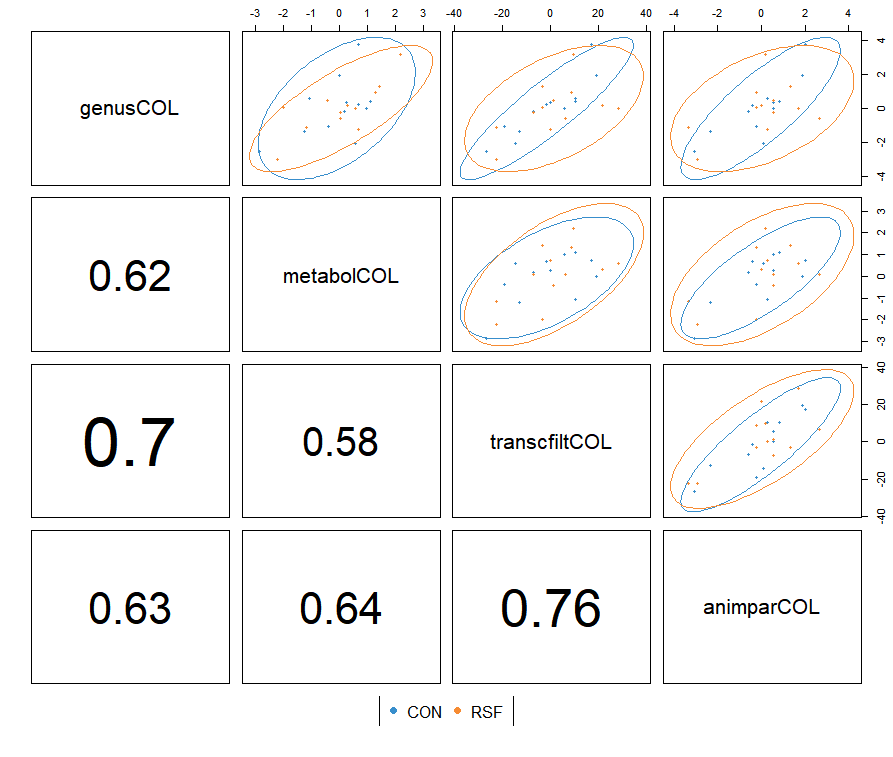


E

**Figure S4.** Diagnostic plot from the Diablo model applied on the colon samples. The samples are represented based on the 1^st^ (A), 2^nd^ (B), 3^rd^ (C), 4^th^ (D) and 5^th^ (E) component of the model. The samples are colored by the diet type and 95% confidence ellipse plots were shown. The numbers indicate the correlation between components from each data set, and the colors and ellipses indicate the discriminative power of each component to separate CON and RSF groups. genusCOL: microbiome data; metabolCOL: metabolome data; transcfiltCOL: transcriptome data; animparCOL: animal parameters data from ileum samples.
